# Sickle haemoglobin, haemoglobin C and malaria mortality feedbacks

**DOI:** 10.1186/s12936-015-1077-5

**Published:** 2016-01-12

**Authors:** Bronner P. Gonçalves, Sunetra Gupta, Bridget S. Penman

**Affiliations:** Department of Immunology and Infection, London School of Hygiene and Tropical Medicine, London, W1CE 7HT UK; Department of Zoology, University of Oxford, South Parks Road, Oxford, OX1 3PS UK

**Keywords:** Malaria, *Plasmodium falciparum*, Sickle cell, Haemoglobin C, Haemoglobinopathies, Human evolution, Gametocytes, Transmission, Sickle haemoglobin

## Abstract

**Background:**

Sickle haemoglobin (HbS) and haemoglobin C (HbC) are both caused by point mutations in the beta globin gene, and both offer substantial malaria protection. Despite the fact that the blood disorder caused by homozygosity for HbC is much less severe than that caused by homozygosity for HbS (sickle cell anaemia), it is the sickle mutation which has come to dominate many old-world malarious regions, whilst HbC is highly restricted in its geographical distribution. It has been suggested that this discrepancy may be due to sickle cell heterozygotes enjoying a higher level of malaria protection than heterozygotes for HbC. A higher fitness of sickle cell heterozygotes relative to HbC heterozygotes could certainly have allowed the sickle cell allele to spread more rapidly. However, observations that carrying either HbC or HbS enhances an individual’s capacity to transmit malaria parasites to mosquitoes could also shed light on this conundrum.

**Methods:**

A population genetic model was used to investigate the evolutionary consequences of the strength of malaria selection being correlated with either HbS frequency or HbC frequency.

**Results:**

If the selection pressure from malaria is positively correlated with the frequency of either HbS or HbC, it is easier for HbS to succeed in the competitive interaction between the two alleles.

**Conclusions:**

A feedback process whereby the presence of variant haemoglobins increases the level of malaria selection in a population could have contributed to the global success of HbS relative to HbC, despite the former’s higher blood disorder cost.

**Electronic supplementary material:**

The online version of this article (doi:10.1186/s12936-015-1077-5) contains supplementary material, which is available to authorized users.

## Background

Falciparum malaria has had a profound effect on human evolution, evidenced by the high frequencies of malaria protective mutations observed in populations from historically malarious regions. Many of the protective variants identified thus far affect erythrocytes, where the malaria parasite spends a crucial stage of its life cycle. Several of the best studied of these mutations affect the globin genes encoding haemoglobin [1]. However, the benefits provided by malaria protective haemoglobin mutations are offset by the fact that such mutations can also lead to genetic blood disorders (haemoglobinopathies).

Haemoglobin C (HbC) is caused by a mutation (henceforth ‘β^C^’) in the 6th position of the amino acid sequence of beta globin, where glutamic acid is substituted by lysine. A recent meta-analysis [2] concluded that homozygotes for β^C^ (genotype CC) were strongly protected against severe malaria, and heterozygotes (genotype AC) were mildly protected. It has also been found that both heterozygotes and homozygotes for β^C^ may be less susceptible to uncomplicated clinical malaria than individuals with the ‘normal’ genotype, AA [3], although a recent cohort study in Mali reports an increase in the incidence of clinical malaria in AC individuals relative to AA [4]. Several mechanisms have been proposed to explain the malaria protection offered by HbC, including: abnormal intraerythrocytic development of the parasite leading to lower *Plasmodium falciparum* replication rates in subsets of CC erythrocytes [5]; abnormal *P. falciparum* erythrocyte membrane protein 1 (PfEMP-1) display, leading to reduced cytoadherence and possibly reduced parasite sequestration [6], and accelerated acquisition of immunity against malaria [7].

Sickle haemoglobin (HbS) is likewise caused by a single amino acid substitution at position 6 of beta globin. The sickle cell mutation (henceforth ‘β^S^’) replaces glutamic acid with valine. Heterozygosity for the sickle mutation (genotype AS) offers considerable protection against all forms of severe malaria, as well as protection against uncomplicated malaria [2] and parasitaemia [8, 9]. The battery of potential protective mechanisms that have been proposed for sickle cell include, like HbC, the abnormal display of PfEMP-1 [10] and the acceleration of acquired immunity [11]. It has also been shown that the growth rate of *P. falciparum* is retarded in HbS containing erythrocytes under conditions of low oxygen tension in vitro [12]; studies in mice have suggested that the protective effect of HbS is lost upon removal of the spleen [13], and an intriguing recent finding is that miRNAs are translocated from erythrocytes to malaria parasites, and specific miRNAs found more commonly in sickle cell trait cells than in normal cells inhibit parasite growth [14].

β^S^ is associated with a high genetic load due to the low fitness of its homozygotes (genotype SS), who suffer from the severe blood disorder sickle cell anaemia. In contrast, the blood disorder associated with the genotype CC is relatively mild [15]. β^C^ frequencies are low in most malaria endemic regions, with the exception of a few locations in West Africa [16], whilst β^S^ is found at frequencies of up to 18 % across sub-Saharan Africa, the middle East and in scattered populations throughout India [17]. These observations create an apparent paradox: why should a malaria protective allele with such minor costs be so limited in its geographical distribution, whilst an allele associated with a severe blood disorder has come to dominate continents?

Recent epidemiological studies have generated the data necessary to estimate the relative malaria protection offered by AC or AS heterozygosity [3, 18]. AC seems to offer lower protection than AS against life-threatening falciparum disease. Furthermore, the genotype SC has been associated with a clinically significant blood disorder, although this condition is less severe than sickle cell disease [19]. It, therefore, seems reasonable to conclude that the relative fitnesses (w) for the genotypes AS, AC and SC have the following relationship w_AS_ > w_AC_ ≫ w_SC_. The low fitness of SC individuals leads to competitive exclusion between β^S^ and β^C^. The fact that AS individuals enjoy more protection against severe malaria than AC individuals [2] means β^S^ will outcompete β^C^ if both are introduced simultaneously in a given population. In a population in Burkina Faso where β^S^ and β^C^ coexist, Modiano et al. [20] showed that β^C^ has been present for approximately four times as long as β^S^. Only by arriving first has β^C^ managed to maintain a relatively high frequency in Burkina Faso, despite the invasion of β^S^.

When considering the factors contributing to the limited geographical distribution of β^C^, Modiano et al. [20] suggest that β^C^ increases in frequency by a predominantly recessive mechanism of selection (since the CC homozygote seems to enjoy more malaria protection than the AC heterozygote), whilst β^S^ spreads much more rapidly through heterozygote advantage. However, there is a further, potentially crucial, feature of the interrelationship between HbS, HbC and *P. falciparum* yet to be considered. Gametocytes (the sexual stage of malaria parasites) are the only form of the parasite that can successfully infect a mosquito taking a blood meal [21]. Host factors, including immune responses, potentially influence gametocyte density during infection [21, 22]. Mutations affecting beta globin have also been associated with different levels of circulating gametocytes and of human infectivity to mosquitoes [23–25]. Ringelhann et al. [24] studied asymptomatic children under five years of age and showed that AC individuals had higher microscopically-detectable gametocyte prevalence than AA individuals. Robert et al. [25] observed that AS gametocyte carriers could infect twice as many mosquitoes as AA gametocyte carriers. Gouagna et al. [23] demonstrated that individuals carrying β^C^ have a higher potential to transmit *P. falciparum* to anopheline vectors than individuals with the genotype AA. Taken together, these studies suggest that the diversity and frequency of beta globin variants could impact local malaria transmission intensity.

In 2004, Feng et al. [26] investigated the possible population genetic consequences of enhanced malaria parasite transmission from sickle cell heterozygotes, showing that such an effect could lead to increased selection for β^S^. In the population genetic model presented here, selection pressure from malaria is linked to either β^S^ or β^C^ frequency, and the consequences of such feedbacks for the competitive relationship between β^S^ and β^C^ are explored.

## Methods

The rate of change in the frequency of each genotype was given by the following equation:

1 $$\frac{{dy_{i} }}{dt} = \varphi g_{i} \left( {p,q} \right) - \left( {\mu_{Bi} + \left( {f_{j} \left( {p,q} \right)} \right)r_{i} } \right)y_{i}$$

where *y*_*i*_ is the frequency of genotype *i* (i = 1–6, where 1 = AA, 2 = AC, 3 = AS, 4 = CC, 5 = SS, 6 = SC); $\varphi$ is the birth rate; *p* is the frequency of β^C^; *q* is the frequency of β^S^, and *g*_*i*_*(p, q)* is a function of *p* and *q* that assigns the proportion of births of each genotype (see Additional file 1 for further details). A non malaria-associated mortality rate (*µ*_*Bi*_), and a level of susceptibility to death from malaria (*r*_*i*_) were estimated for each genotype based on their known properties (see Additional file 1: Tables S1 and S2). The maximum possible malaria mortality rate was made a function of β^C^ or β^S^ frequencies by *f*_*j*_*(p, q).* If malaria mortality was positively correlated with allele frequency, function *f*_*1*_ was applied (Eq. 2); if malaria mortality was negatively correlated with the frequency of an allele, function *f*_*2*_ was applied (Eq. 3).

2 $$f_{1} \left( {p,q} \right) = \hbox{min} \left( {x\text{ + }\gamma u\left( {z\text{ - }x} \right),z} \right)$$

3 $$f_{2} \left( {p,q} \right) = \hbox{max} \left( {z\text{ - }\gamma u\left( {z\text{ - }x} \right),x} \right)$$

*x* is the minimum level of malaria related mortality assumed possible; *z* is the maximum level of malaria related mortality assumed possible, and *γ* is a coefficient scaling the effect of allele frequency on the level of mortality due to malaria. *u* may be equal to the frequency of the sickle cell allele (*q*) or the HbC allele (*p*).

The population size was set to be constant (limited by an external carrying capacity) by making the birth rate equal to the total death rate (Eq. 4):

4 $$\varphi = \sum\limits_{i = 1}^{6} {\left( {\mu_{Bi} \text{ + }\left( {f_{j} \left( {p,q} \right)} \right)r_{i} } \right)y_{i} }$$

## Results

### A positive correlation between malaria mortality and either β^S^ or β^C^ frequency favours β^S^ at equilibrium

Specific conditions must be met if stable coexistence between three alleles is to be achieved, and, given realistic assumptions about each genotype’s relative susceptibility to severe malaria and blood disorder costs, stable coexistence between β^S^, β^C^ and β^A^ is not possible [27]. There will always be a “winner” in the competition between β^S^ and β^C^, and, with the mortality rates used here, if both alleles are seeded in a population at equal frequencies, β^S^ will always eliminate β^C^.

**Fig. 1:**
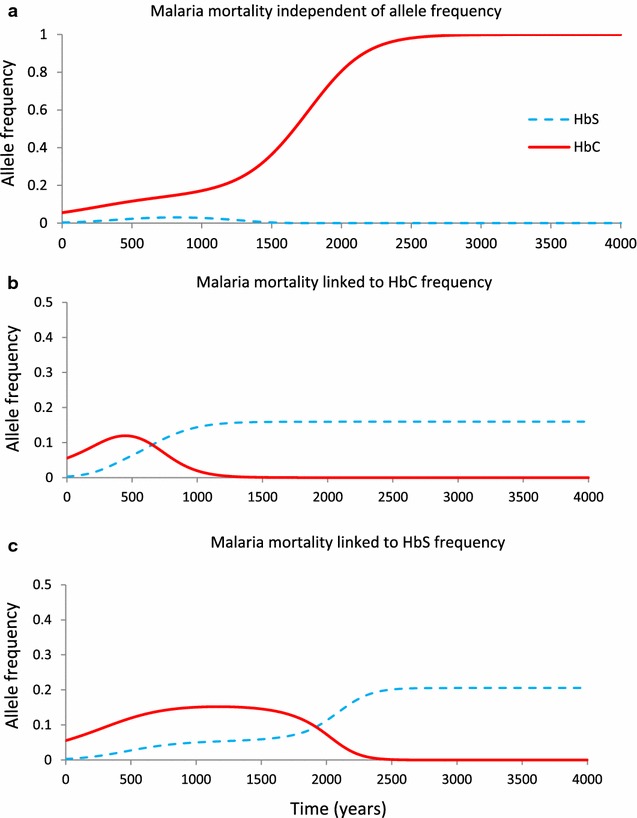
Dynamics of β^C^ and β^S^ frequencies under different malaria mortality functions. In each simulation shown, β^C^ starts at a frequency of 5.6 % and β^S^ starts at a frequency of 0.3 %, equivalent to a single β^S^ heterozygote in a population of 150 individuals (e.g., after a mutation arises de novo or is imported into a small population). The malaria mortality function applied in all* panels* is *f*_*1*_, where *x* = 0.01 and *z* = 0.015. In **a**, *γ* = 0, thus malaria mortality is independent of allele frequency. In **b**, *γ* = 10 and malaria mortality increases with β^C^ frequency (*u* = *p*); in **c**, *γ* = 10 and malaria mortality increases with β^S^ frequency (*u* = *q*). Baseline mortality rates and malaria susceptibilities of each genotype are given in tables S1 and S2

Figure 1 explores a scenario in which β^C^ is present in a population prior to the arrival of β^S^, as seems to have occurred in West Africa. If β^C^ is at a threshold frequency before the introduction of β^S^, β^C^ will be able to prevent β^S^ from successfully invading (Fig. 1a). However, if the cost of malaria increases linearly with the frequency of β^C^ (Fig. 1b), then even with exactly the same starting conditions as in panel a, there will come a point where the strength of malaria selection is such that β^S^ can eliminate β^C^. If malaria mortality is linked to β^S^ frequency, β^S^ will still replace β^C^, but it will take longer and the frequency of β^S^ at equilibrium will be higher (Fig. 1c).

**Fig. 2:**
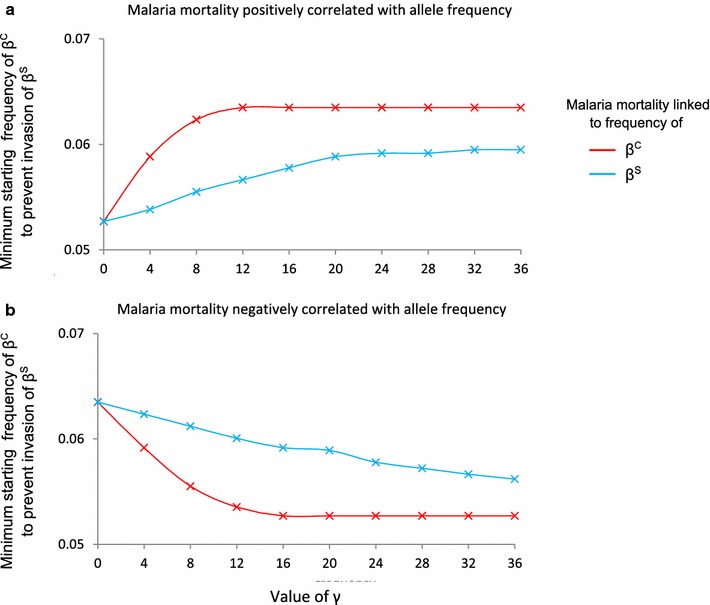
How the threshold starting frequency of β^C^ required to prevent the invasion of β^S^ varies with different malaria mortality functions. The starting frequency of β^S^ was always 0.3 %. β^S^ was deemed to have failed to successfully invade the population if its frequency (*q*) was <(1/300) after 15,000 years of evolution. Each panel shows how the minimum starting frequency of β^C^ required to prevent β^S^ invading varies with different values of *γ* (*x axis*). In **a**, function *f*_*1*_ has been applied; in **b**, function *f*_*2*_ is used. In both* panels*, the *red line* indicates the case where malaria mortality is linked to β^C^ frequency (*u* = *p*) and the *blue line* indicates the case where malaria mortality is linked to β^S^ frequency (*u* = *q*). Baseline mortality rates and malaria susceptibilities of each genotype are given in tables S1 and S2

Figure 2 illustrates the minimum initial frequency of β^C^ required to prevent its elimination by β^S^ under different conditions. If β^C^ acts to increase malaria mortality in a population, the threshold frequency increases with the intensity of this effect (Fig. 2a, red line). If malaria mortality increases with β^S^ frequency, the threshold initial frequency also increases with the intensity of the effect, but not so steeply (Fig. 2a, blue line).

In Fig. 1 and Fig. 2a, higher frequencies of β^S^ and β^C^ are assumed to be associated with increasing malaria mortality. However, the incidence of severe malaria during childhood may be inversely related to transmission intensity. Snow et al. [28] showed that the risk of severe malaria declines between low-to-moderate and high malaria transmission intensity regions, perhaps because in a highly endemic area, individuals are infected very early in their lives and build up protective immunity whilst shielded by maternal antibodies. It is therefore conceivable that by increasing malaria transmission, higher frequencies of β^S^ or β^C^ could result in lower overall malaria mortality. A positive relationship between beta globin mutation frequencies and malaria mortality benefits the ‘more protective yet costly’ allele (β^S^). A negative relationship between beta globin mutation frequencies and malaria mortality, benefits the ‘less protective yet mild’ allele (β^C^), as illustrated in Fig. 2b.

### Linking malaria selection pressure to allele frequency dramatically alters the fitness landscape

**Fig. 3:**
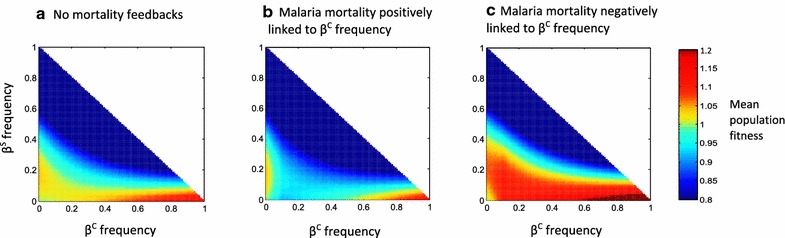
The combined effect of β^C^ and β^S^ frequencies on overall population fitness. The colours in each panel indicate the mean fitness of a population with a specific combination of β^C^ frequency (*x*-*axis*) and β^S^ frequency (*y*-*axis*), relative to the fitness of an AA individual in a population of only AA individuals. The malaria mortality function applied in **a** is *f*_*1*_, where *x* = 0.01, *z* = 0.015 and *γ* = 0; the malaria mortality function applied in **b** is *f*_*1*_, where *x* = 0.01, *z* = 0.015 and *γ* = 10, and the malaria mortality function applied in **c** is *f*_*2*_, where *x* = 0.01, *z* = 0.015 and *γ* = 10. In all three panels, *u* = *p*

Figure 3 illustrates the effects of different combinations of β^C^ and β^S^ frequencies on the mean fitness of a population. If the presence of β^C^ amplifies malaria mortality, there is a critical zone where increasing β^C^ frequency, in the presence of a low frequency of β^S^, *decreases* the mean fitness of the population. In this zone, β^S^ will clearly be favoured—hence an increased likelihood of β^S^ dominating the population. By contrast, when β^C^ frequency is negatively correlated with malaria mortality, increasing β^C^ frequency in the presence of a low level of β^S^ always *increases* the mean fitness of the population. In this fitness landscape, it is harder for β^S^ to displace β^C^.

## Discussion

These results demonstrate that a link between the frequency of β^C^ or β^S^ and the strength of malaria selection can change the outcome of the competitive interaction between these two malaria protective alleles. If there is a positive relationship between β^C^ or β^S^ frequencies and malaria selection, then the allele that offers more protection in the heterozygous state (β^S^) will be favoured.

Both β^S^ and β^C^ are point mutations. However, β^S^ is caused by a A→T mutation (a transversion from a purine to a pyrimidine), whilst β^C^ is caused by a G→A mutation (a transition between purines). Transitions occur at a higher rate than transversions [29]: the β^C^ substitution should, therefore, arise spontaneously at a higher rate than the β^S^ substitution. Given this discrepancy, the relative absence of β^C^ in malarious regions is all the more surprising. Even if, as Modiano et al. pointed out [20], β^C^ needs a longer time to establish itself, should it not have had more opportunities to do so? An increase in malaria-related selection pressure with increasing β^C^ or β^S^ frequencies could be precisely the mechanism required to account for the preponderance of β^S^. Furthermore, the behaviour exhibited by the population genetic framework presented here includes the elimination of β^C^ even after it reaches a relatively high frequency. It is possible, therefore, that β^C^ dominated a much larger area of West Africa historically before β^S^ arrived.

Gametocytogenesis in *P. falciparum* is still poorly understood [30], so the processes underlying increased gametocyte prevalence in the presence of β^C^ [24] and β^S^ [31] are unknown. It is possible that the presence of haemoglobin S or HbC is associated with a harsh environment for parasite development, and that this stimulates *P. falciparum* to commit to the sexual stage. Previous studies have suggested that rodent and avian malaria parasites’ transmission-related phenotypes (conversion rate and sex ratio) change in response to a deterioration of the blood environment [32–34]. Trager et al. [35] have shown that *P.falciparum* parasites formed up to seven times more gametocytes in reticulocyte-rich blood from individuals with sickle cell anaemia compared to control blood. Understanding this phenotypic plasticity is a central part of understanding the transmission biology of malaria parasites.

The relationship between clinical severity and transmission potential during malaria infections is far from clear, and the model presented here was not intended to explore this question. Hypothetically, however, if (i) severe disease is caused by a subset of particularly virulent parasite strains, e.g., strains with cytoadherence properties that favour sequestration in vital organs, and (ii) virulence is associated with increased transmission, as has been demonstrated for rodent malarias [36], it is conceivable that the very presence of HbS and HbC in a population could encourage the circulation of more virulent and transmissible parasites. Gupta & Hill have shown that the presence of a host allele that confers resistance to death from infection can increase the likelihood of a virulent (and more transmissible) pathogen strain persisting, despite competition from less virulent strains [37]. The presence of inherently more virulent and transmissible parasites in populations containing either HbS or HbC could be a further factor increasing malaria selection wherever HbS or HbC are spreading. As shown by the results presented here, any increase in malaria selection under such circumstances favours the ultimate success of HbS over HbC.

The framework presented here demonstrates a point of principle. There are two obvious ways in which this work can be extended: (i) the incorporation of parasite strains that differ in their virulence and transmissibility, enabling the consequences of feedbacks between β^C^, β^S^, virulence and transmissibility to be explored; (ii) the extension of this model into a metapopulation framework, which would allow the inclusion of spatial heterogeneities in malaria selection pressure. Further studies are also needed to place the observed effects of haemoglobin variants on malaria transmissibility [23] into their full epidemiological context. Studies controlling for factors influencing transmission intensity (e.g., the spatial and temporal distribution of anopheline mosquitoes) and comparing malaria infection rates, malaria mortality rates and β^C^ and β^S^ frequencies in different communities will help to establish the strength and direction of the effects of β^C^ and β^S^ on malaria dynamics in different transmission environments.

## Conclusions

The population genetic model presented here demonstrates that a positive relationship between the frequency of either β^C^ or β^S^ and malaria selection intensity favours the predominance of β^S^. Recent observations that both of these mutations are associated with increased malaria transmission potential [23–25, 31] may, therefore, help to account for the fact that β^S^ attains high frequencies throughout sub Saharan Africa, whilst high frequencies of β^C^ are limited to parts of West Africa.

In the long term, understanding the mechanisms underlying the influence of β^C^ and β^S^ on gametocyte levels and infectivity will provide important insights not only for understanding the evolutionary genetics of malaria but also for public health. If higher frequencies of β^C^ and β^S^ enhance transmission, targeted transmission blocking strategies in populations prone to infect more mosquitoes could be part of malaria intervention programmes.

## Additional file

10.1186/s12936-015-1077-5 Supplementary Methods and Supplementary Tables.
