# Supplementary material for: Sickle haemoglobin, haemoglobin C and malaria mortality feedbacks
Source: Malar J. 2016 Jan 12;15:26. doi: 10.1186/s12936-015-1077-5 (PMC4709991; doi:10.1186/s12936-015-1077-5)
Supplement: Supplementary file 1 — 10.1186/s12936-015-1077-5 Supplementary Methods and Supplementary Tables. [file 12936_2015_1077_MOESM1_ESM.doc]

**Supplementary methods:**

Allele frequencies *p* and *q* were calculated as follows:

$p=\frac{2y_{4}+y_{2}+y_{6}}{2\sum_{i=1}^{6} y_{i}}$ $q=\frac{2y_{5}+y_{3}+y_{6}}{2\sum_{i=1}^{6} y_{i}}$

Functions *g_1_-g_6_* were as follows (i=1-6, where 1=AA, 2=AC, 3= AS, 4=CC, 5 = SS, 6 =SC):

$g_{1}(p,q)=(1-p-q)^{2}$

$g_{2}(p,q)=2(1-p-q).p$

$g_{3}(p,q)=2(1-p-q).q$

$g_{4}(p,q)=p^{2}$

$g_{5}(p,q)=q^{2}$

$g_{6}(p,q)=2pq$

**Supplementary Tables**

**Table S1: Non malaria associated mortalities (μ_B_) used in our model. Genotypes AC, CC and AS were assumed to have no excess mortality associated with their abnormal haemoglobin and hence were assigned a non-malaria-associated mortality rate equal to that of genotype AA. Hemoglobin SC disease is a life threatening disorder. Although hemolysis is less severe compared to SS individuals, the life span in those individuals is shorter than AA individuals (Nagel et al, 2003). We consider that, historically, having either genotype SS or SC will have substantially impacted an individual’s chances of surviving and reproducing – hence the higher mortality rates assigned to these genotypes.**

| *Genotypes* | *HbAA* | *HbAC* | *HbCC* | *HbAS* | *HbSS* | *HbSC* |
| --- | --- | --- | --- | --- | --- | --- |
| Mortality  (years^-1^) | 0.04 | 0.04 | 0.04 | 0.04 | 1 | 0.80 |

**Table S2: Malaria Susceptibilities used in our model.** Genotype AA was assumed to confer no protection against death from malaria (susceptibility = 1). SS individuals do not appear to be protected against severe malarial disease (McAuley *et al* 2010), so we also assigned a malaria susceptibility of 1 to this genotype,although in the context of this model, the severity of the blood disorder associated with SS will have a much more important impact than any malaria advantage or disadvantage. For the relative susceptibilities of each of the other genotypes we have assigned plausible values based on their reported protectiveness against severe malaria. Severe malaria incidence rate ratios are not available for all genotypes, so for consistency we have used reported odds ratios. A meta analysis performed by Taylor *et al* calculates the overall odds ratio of AC versus AA individuals developing severe malaria to be 0.80 (95% CI= 0.67-0.96); the odds ratio of CC versus AA individuals developing severe malaria to be 0.27 (95% CI= 0.11-0.63) and the odds ratio of AS versus AA individuals developing severe malaria to be 0.09 (95% CI= 0.06-0.12). A case-control study in Ghana (May *et al* 2007) estimated the odds ratio of SC versus AA children developing severe malaria to be 0.44 (95% CI= 0.18-1.04).

| *Genotypes* | *HbAA* | *HbAC* | *HbCC* | *HbAS* | *HbSS* | *HbSC* |
| --- | --- | --- | --- | --- | --- | --- |
| Relative susceptibility to death from malaria | 1 | 0.80 | 0.27 | 0.09 | 1 | 0.44 |

**References**

May, J., Evans, J.A., Timmann, C., Ehmen, C., Busch, W., Thye, T., Agbenyega, T., Horstmann, R.D., 2007. Hemoglobin Variants and Disease Manifestations in Severe Falciparum Malaria. JAMA 297(20), 2220-2226

# McAuley C.F., Webb C., Makani J., Macharia A., Uyoga S., Opi D.H., Ndila C., Ngatia A., Scott J.A., Marsh K., Williams T.N., 2010. High mortality from Plasmodium falciparum malaria in children living with sickle cell anemia on the coast of Kenya. Blood 116(10): 1663-8

Nagel, R.L., Fabry, M.E., Steinberg, M.H., 2003.The paradox of hemoglobin SC disease. Blood Rev 17(3), 167-178

Taylor, S.M., Parobek, C.M., Fairhurst, R.M, 2012. Haemoglobinopathies and the clinical epidemiology of malaria: a systematic review and meta-analysis. Lancet Infect Dis.,12(6), 457-68.
